# Recurrent evolution of host and vector association in bacteria of the *Borrelia burgdorferi* sensu lato species complex

**DOI:** 10.1186/s12864-016-3016-4

**Published:** 2016-09-15

**Authors:** Noémie S. Becker, Gabriele Margos, Helmut Blum, Stefan Krebs, Alexander Graf, Robert S. Lane, Santiago Castillo-Ramírez, Andreas Sing, Volker Fingerle

**Affiliations:** 1 Faculty of Biology, Division of Evolutionary Biology, Ludwig Maximilians University of Munich, Grosshaderner Strasse 2, 82152 Planegg-Martinsried, Germany; 2 National Reference Centre for Borrelia at the Bavarian Health and Food Safety Authority, Veterinärstr. 2, 85764 Oberschleissheim, Germany; 3 Gene Center, Laboratory for Functional Genome Analysis, Ludwig Maximilians University of Munich, Feodor-Lynen-Strasse 25, 81377 Munich, Germany; 4 Department of Environmental Science, Policy and Management, University of California, Berkeley, CA USA; 5 Programa de Genómica Evolutiva, Centro de Ciencias Genómicas, Universidad Nacional Autónoma de México, Apartado Postal 565-A, CP 62210 Cuernavaca, Morelos Mexico

**Keywords:** Borrelia burgdorferi sensu lato, Evolution, Population genomics, Host association, Vector adaptation, Lyme disease

## Abstract

**Background:**

The *Borrelia burgdorferi* sensu lato (s.l.) species complex consists of tick-transmitted bacteria and currently comprises approximately 20 named and proposed genospecies some of which are known to cause Lyme Borreliosis. Species have been defined via genetic distances and ecological niches they occupy. Understanding the evolutionary relationship of species of the complex is fundamental to explaining patterns of speciation. This in turn forms a crucial basis to frame testable hypotheses concerning the underlying processes including host and vector adaptations.

**Results:**

Illumina Technology was used to obtain genome-wide sequence data for 93 strains of 14 named genospecies of the *B. burgdorferi* species complex and genomic data already published for 18 additional strain (including one new species) was added. Phylogenetic reconstruction based on 114 orthologous single copy genes shows that the genospecies represent clearly distinguishable taxa with recent and still ongoing speciation events apparent in Europe and Asia. The position of *Borrelia* species in the phylogeny is consistent with host associations constituting a major driver for speciation. Interestingly, the data also demonstrate that vector associations are an additional driver for diversification in this tick-borne species complex. This is particularly obvious in *B. bavariensis,* a rodent adapted species that has diverged from the bird-associated *B. garinii* most likely in Asia. It now consists of two populations one of which most probably invaded Europe following adaptation to a new vector (*Ixodes ricinus*) and currently expands its distribution range.

**Conclusions:**

The results imply that genotypes/species with novel properties regarding host or vector associations have evolved recurrently during the history of the species complex and may emerge at any time. We suggest that the finding of vector associations as a driver for diversification may be a general pattern for tick-borne pathogens. The core genome analysis presented here provides an important source for investigations of the underlying mechanisms of speciation in tick-borne pathogens.

**Electronic supplementary material:**

The online version of this article (doi:10.1186/s12864-016-3016-4) contains supplementary material, which is available to authorized users.

## Background

High-throughput sequencing of whole genomes of bacterial pathogens enables phylogenetic analysis of the core genome. Phylogenetic analysis is a powerful tool for generating hypotheses on the pattern of descent and evolution of species and strains, their host and vector adaptation or pathogenicity. This in turn provides information for comparative genomics, an effective approach for studying genetic differences between strains and/or species that may lead to differences in phenotype related to host or vector associations, pathogenicity and others [1, 2]. In this study we used vector-borne bacterial pathogens, the *Borrelia burgdorferi* sensu lato (s.l.) species complex, to advance the understanding of the phylogenetic relationship of members of this species complex and to put into perspective the evolution of vector- and host associations.

*Borrelia burgdorferi* s.l., also termed the Lyme Borreliosis (LB) group of spirochetes, forms a species complex now comprising about 20 named and proposed genospecies [3]. The parasitic bacteria are maintained in natural transmission cycles between reservoir hosts and tick vectors of the genus *Ixodes* [4]. A hallmark in the ecology of *Borrelia* is the association with vertebrate reservoir hosts which have been shown to have a major impact on the population structure [5–7]. Some species occupy wide niches being able to utilize a large range of reservoir hosts spanning several species orders (e.g., *Borrelia burgdorferi* sensu stricto) while others (such as *B. spielmanii*, *B. lusitaniae* or *B. californiensis*) have a narrow reservoir host range [8–11] and implications on the geographic distribution and dispersal of the bacteria have been suggested [12, 13]. Similarly, some *Borrelia* species are transmitted by *Ixodes* species which are considered generalist feeders, e.g., *Ixodes ricinus, I. scapularis, I. persulcatus,* while others are adapted to tick species with a narrow host preference such as *I. spinipalpis, I. minor* or *I. ovatus* (reviewed by [14–16]). Both, host and vector associations are obviously contributing to the asymmetrical geographic distribution of species within the distribution range between around 40th and 60th degree northern latitude but the question of how these traits evolved has remained unresolved.

Studies on the phylogenetic relationship and global evolution of the *B. burgdorferi* s.l. species complex were performed using MultiLocus Sequence Analysis (MLSA) or genomic data [17, 18]. As expected when using a limited number of loci (as used for MLSA) support of internal nodes was low and therefore unreliable [17]. Whole genome data were so far available only for few species of the complex and it is well known that addition of more taxa or more loci may provide more accurate phylogenies [19, 20]. A more comprehensive study on *B. burgdorferi* sensu lato was conducted by Mongodin et al. [21] which included eight species of the complex and showed that a robust reconstruction of the evolutionary relationship was obtained using genome-wide single nucleotide polymorphisms (SNPs).

The aim of our study was to produce a robust phylogeny for the *B. burgdorferi* s.l. species complex to obtain a better understanding of their global evolution and to put into perspective the evolution of host and vector adaptations. Using Illumina technology we sequenced 93 strains belonging to 14 previously described genospecies of the *B. burgdorferi* s.l. species complex. In addition we downloaded from GenBank [22] chromosome sequence data for 18 samples including one belonging to a species *(B. chilensis)* not present in our own dataset. We applied a Bayesian method to reconstruct a phylogeny with high internal node support based on 114 orthologous single copy genes. Our data provide evidence that neither host nor vector adaptations cluster monophyletically suggesting that they developed several times independently during the evolutionary history of the complex. Adaptation either to a new host or a new vector probably lead to ecological isolation and hence to speciation. The NGS tree suggests that both processes have been important in the evolutionary history of *Borrelia* spirochetes.

## Results

To gain knowledge on the global evolution of the bacterial species complex *B. burgdorferi* s.l., we sequenced 93 strains belonging to 14 of the approximately 20 described genospecies of the complex (Additional file 1: S1) and downloaded chromosome sequence data for 18 additional LB strains including one strain of *B. chilensis* [23] and for the Relapsing Fever strain Ly of *B. duttonii* that was used as outgroup in the phylogeny [24]. Our final dataset thus comprised 111 LB strains from 15 species and 1 outgroup strain. For 11 of the species between one and three strains were sequenced. For four species, a large number of isolates was available and our final dataset contains 16 *B. afzelii*, 29 *B. bavariensis*, 22 *B. burgdorferi* s.s. and 26 *B. garinii*. This allowed us to get an impression on intraspecific variation. *B. afzelii* and *B. garinii* are the most frequently found species in questing *I. ricinus* in Europe and all four species are also frequently found in human patients.

### Phylogenetic analyses of single copy orthologous loci

**Fig. 1:**
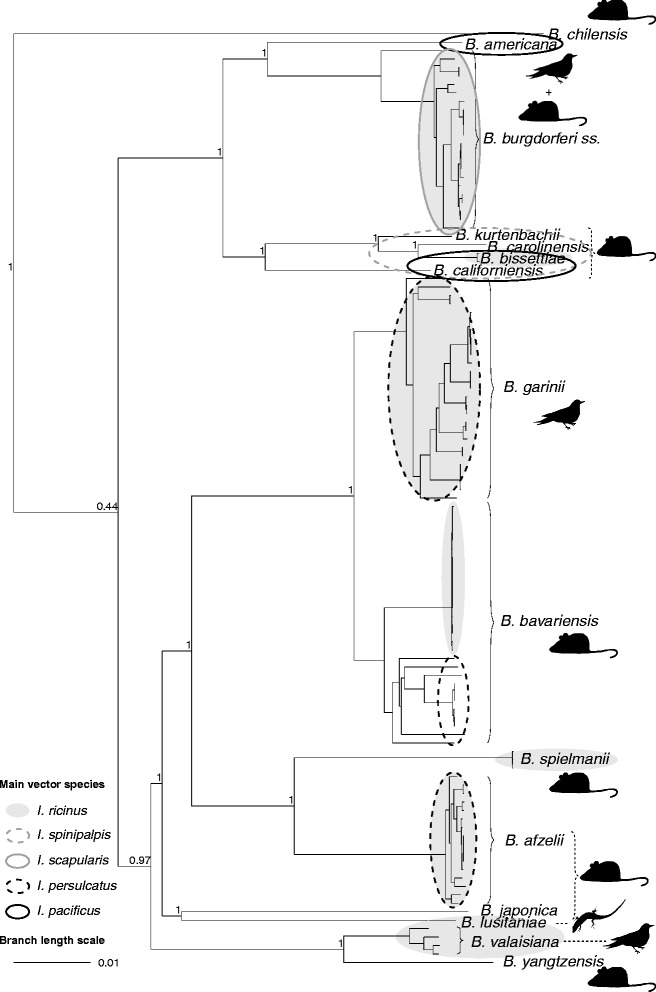
Phylogenetic inference and main host and vector association of *B. burgdorferi* sensu lato The phylogeny reconstructed with BEAST v1.7.5 [26] is based on 114 orthologous single copy genes. Genospecies names and host associations are indicated next to the cluster. As huge number of host species can serve as reservoirs for *Borrelia* [4], for sake of clarity, we used a crude host association referring to rodents (indicated by the mouse), birds (indicated by the black bird) and lizards. For the same reason, not all vector associations can be shown. The following vectors are not shown in the phylogeny (which does not mean that they are less important for natural transmission cycles): *I. hexagonus* (vector of *B. afzelii*, *B. burgdorferi* s.s., *B. bavariensis*), *I. affinis* (*B. burgdorferi* s.s., *B. bissettiae*), *I. minor* (*B. americana*, *B. carolinensis*), *I. jellisoni* (*B. californiensis*), *I. uriae, I. pavlovskyi* (*B. garinii*), *I. stilesi* (*B. chilensis*) and *I. granulatus* (*B. yangtzensis*) and *I. nipponensis*. Note the high posterior probability of internal nodes (within-species node posterior probabilities are not shown for sake of clarity). The scale bar indicates substitutions per site

Using blastn [25] searches 114 orthologous single copy loci of sufficient quality were found in all LB strains and were included in phylogenetic analyses. Using these data a robust phylogeny was reconstructed in BEAST v1.7.5 [26] where known *Borrelia* species are separated by long branches with good posterior probabilities of inner nodes (Fig. 1). After splitting of *B. chilensis* strain VA1, the phylogeny is composed of two sister clades – one clade that contains the species known from Europe and Asia; the other one consists of species that are known to occur in North America (with *B. burgdorferi* s.s. and *B. bissettiae* also occurring in Europe). The root of the tree inferred by BEAST [26] is confirmed by a phylogeny reconstructed using 37 orthologous genes for the LB strains and the outgroup *B. duttonii* strain Ly [24] where *B. chilensis* is also the first species to diverge and where the posterior probability of the node separating the “Eurasian” and “American” clades is much more satisfactory (1 instead of 0.44). This second phylogeny, presented in Additional file 1: S2, is extremely similar to the first one except for the position of *B. lusitaniae* and *B. japonica* that cluster with other species instead of forming a clade together. This small discrepancy is probably due to the reduced power of the analysis with only 37 genes found orthologous with the outgroup.

In the phylogeny shown in Fig. 1, *Borrelia* strains generally cluster according to species except strain PKl. It is likely that the strain in our analysis was mislabeled and that the strain analyzed here was *B. garinii* strain PKi. This sample was not included in the estimation of species-specific summary statistics. Three strains identified as *B. garinii* in GenBank [22] and BorreliaBase [27]: BgVir strain from Russia [28] and SZ and NMJW1 strains from China [29, 30] clustered within the *B. bavariensis* clade and were considered as *B. bavariensis* in our analyses. We had already shown in a previous study [31] that strains BgVir and NMJW1 clustered within *B. bavariensis* and we suggest that all three strains require re-classification.

In the clade containing species from Europe and Asia, *B. spielmanii* and *B. afzelii* form sister clades that seem to have recently diverged. Similarly, *B. bavariensis* and *B. garinii* are well diverged from one another by a recent speciation event likely induced by a host switch (bird or rodent; see Fig. 1). The two populations of *B. bavariensis*, the European and Asian population, are also very well separated. *B. lusitaniae* and *B. japonica* are separated by long terminal branches while *B. valaisiana* branch off together with *B. yangtzensis* suggesting a host switch (from bird to rodent or vice versa) as driver for speciation. This bears remarkable similarity to the speciation event separating *B. garinii* and *B. bavariensis.*

The “American clade”, is separated into two clusters, one that includes *B. bissettiae*, *B. carolinensis*, *B. kurtenbachii* and *B. californiensis,* and one that includes *B. americana* and *B. burgdorferi* s.s.. Thus *B. burgdorferi* s.s. is not representative for the whole species complex, not even the “American” clade even if our dataset contained *B. burgdorferi s.s.* strains from Europe and USA (Fig. 1 and Additional file 1: S1).

There was no monophyletic pattern discernible for vector or host association, i.e., *Borrelia* species being adapted to rodents or birds did not cluster monophyletically (i.e., have a common ancestor) (Fig. 1). For example, both *B. garinii* and *B. valaisiana* utilize birds as reservoir hosts but fall into different clades of the tree separated by clades of rodent associated *Borrelia* species such as *B. afzelii* and *B. spielmanii*. Similarly, rodent adapted species are separated in the phylogeny by *Borrelia* species utilizing different reservoir hosts (lizards, birds) (Fig. 1). The same is true for vector association: not all species that are associated with *I. ricinus* form a monophyletic cluster (Fig. 1). For example, *B. burgdorferi* s.s. is transmitted by *I. ricinus* as most European species (such as *B. garinii*, *B. valaisiana* or *B. afzelii*) but clusters far apart from those species. These data suggest that switches to different hosts and vectors have occurred several times independently throughout the evolutionary history of the species complex.

Inferences about human pathogenicity are more difficult because it is unknown whether species that are maintained in specific enzootic transmission cycles are actually human pathogenic or not, i.e., they are not being found in human patients because they are associated with vectors that do not bite humans at any frequency. A species that is unlikely to be human pathogenic is *B. valaisiana;* it occurs at sufficiently high prevalence in Europe and is associated with a vector that is known to bite humans but *B. valaisiana* strains have never been isolated from a human patient.

### Divergence of clades

The branch length estimates in phylogenies using BEAST [26] are expressed as number of substitutions per site. By multiplying branch length by a mutation rate of 10^7^ substitutions per site per generation (a mutation rate previously used by [20]) we obtained rough estimates for the divergence of different clades. First, using the phylogeny rooted with the outgroup *B. duttonii*, we estimated a divergence time of the LB group with *B. duttonii* of approximately 1,5 million generations. Second, we used branch times within the phylogeny without outgroup (to prevent a potential difference in mutation rate between the outgroup and the LB *Borrelia* to influence our estimates) to estimate the following: the divergence between *B. garinii* and *B. burgdorferi* s.s. was estimated to have occurred 450,000 generations ago (490,000 generations in ref [20]). The split between *B. garinii* and *B. bavariensis* was estimated to have occurred around 137,000 generations ago and between the two *B. bavariensis* populations 92,000 generations ago. Rough estimates of divergence times from the clade regrouping *B. burgdorferi* s.s. and species occurring in North America can be found in Additional file 1: S3. It is important to note that these estimates depend directly on the chosen mutation rate which has not been proven to be accurate for *B. burgdorferi* s.l..

### Clonal Frame ML analysis

We ran ClonalFrameML [32] to estimate the relative impact of mutation and recombination on the phylogeny. This analysis showed that recombination occurred within and between species. Mean ratios of *R/θ* were estimated to be 0.182 suggesting that mutation remains the leading force of evolution for the core genome of *Borrelia* that was investigated here.

### Summary statistics

Genetic diversity (*π*) [33] andTajima’s *D* [34] were computed for the four species with sufficient numbers of strains (i.e., *B. burgdorferi* s.s., *B. garinii*, *B. bavariensis* and *B. afzelii*). In general, the genetic diversity (*π*) was found to be marginally higher in *B. garinii* and *B. bavariensis* (0.007 and 0.008 respectively) than in *B. burgdorferi* s.s. (0.004) and *B. afzelii* (0.002) (Table 1). The mean Tajima’s *D* was negative for all populations, in particular for *B. burgdorferi* s.s suggesting population expansions (Table 1).

**Table 1:** Mean values (standard deviation) of nucleotide diversity (*π*) [33] and Tajima’s *D* [34] for 114 orthologous genes computed using R package pegas on all samples and within *Borrelia* genospecies

	Diversity *π*	Tajima’s *D*
All samples (111)	0.045 (0.011)	−0.13 (0.40)
*B. garinii* (26)	0.007 (0.003)	−0.48 (0.59)
*B. bavariensis* (29)	0.008 (0,004)	−0.60 (0.72)
*B. burgdorferi s.s.*(22)	0.004 (0.003)	−1.21 (0.65)
*B. afzelii* (16)	0.002 (0.001)	−0.42 (0.85)

Additional file 1: S4 shows genes with *π* or Tajima’s *D* values that deviate by more than two standard deviations from mean values. Low values are underlined and high values are in bold letters. These loci could potentially be important during speciation events or during divergence of *Borrelia* populations.

Five genes are present in more than one list of genes showing extreme values within a species. In particular we can highlight three genes that could be under balancing selection. (i) The gene of the ribosomal protein *rplL* (BB0390) has a high Tajima’s *D* in *B. garinii* and in *B. afzelii*. (ii) The cytidine deaminase gene *cdd* (BB0618) has a high diversity and a high Tajima’s *D* in the two sister species *B. garinii* and *B. bavariensis*. (iii) The chemotaxis response regulator BB0570 has high diversity in *B. burgdorferi ss* and *B. afzelii* as well as high Tajima’s D in *B. afzelii*. In general, the fact that most of these genes showing extreme values are not shared between species might indicate that the different species have undergone divergent evolution.

All the genes identified here deserve further analysis within species with more samples in order to determine whether they might have been the target of natural selection as shown in a previous paper for *B. bavariensis* [31].

## Discussion

### Host and vector-association as drivers of the evolution of the *B. burgdorferi* s.l. species complex

We investigated the global evolution of the *B. burgdorferi* s.l. species complex based on sequences of 15 genospecies. For four species (*B. burgdorferi* s.s., *B. afzelii*, *B. garinii*, *B. bavariensis*) enough strains were available for us to look at intraspecies variation.

In the phylogeny resulting from 114 single copy orthologous genes located on the main chromosome (representing the core genome) strains generally clustered according to previously determined genospecies and these formed well separated terminal clades suggesting that they are species-like fundamental units [35]. Including additional members of species that were represented by only one strain might improve the branch length estimation but will probably not change the topology as the genospecies are clearly separated. The robustness of the inferred phylogeny is established by the high statistical support of the internal nodes, in particular when compared to previous analyses using multilocus sequences typing (MLST) (e.g., [3, 36, 37]). In all MLST trees, independent of the tree building method used, statistical support of internal nodes is too low to draw definite conclusions on the evolutionary relationship amongst species and thus, this is a major improvement in our tree. Although the overall topology is very similar between MLST trees, there are slight differences due to different taxa being included (e.g., inclusion of *B. mayonii* and *B. chilensis*, exclusion of *B. yangtzensis* [37] and vice versa [3]). Slight inconsistencies between our tree and the MLST trees may relate to the same fact. In all trees, *B. spielmanii* and *B. afzelii* cluster close together, the same is true for *B. bavariensis* and *B. garinii*. Whenever *B. yangtzensis* is included, it forms a sister clade to *B. valaisiana*. The addition of taxa (example outgroup in Additional file 1: S2) changes slightly the clustering of *B. lusitaniae* and *B. japonica* but such changes are to be expected when additional taxa are included.

It has been suggested that host association is a major driver in *Borrelia* diversification (reviewed by [13, 38]) and our data are consistent with it, i.e., host association appears to be one of the underlying ecological principles of speciation: lineages associated with certain reservoir hosts form terminal clades. The clustering according to genospecies was also shown by other studies employing genome sequences for *Borrelia* species from a limited geographic region [20] or a limited number of species [18].

Our data show, that diversification of lineages may also be driven by vector association. This is particularly obvious in the genospecies *B. bavariensis*: there are two populations of *B. bavariensis*, a heterologous population that occurs in Asia and a genetically homogeneous population that occurs in Europe. The former is vectored by *I. persulcatus* [39–41] while the latter occurs within the distribution range of *I. ricinus* [41–43]. The genetic homogeneity of the European population resembles a population that has founded a new ecotype after a strong bottleneck [44]. This has led to the hypothesis that the European population has arisen via adaptation to a new vector [31]. While both populations are genetically sufficiently closely related to clearly belong to the same species, further divergence and ultimately speciation is predictable through ecological isolation due to different vector associations.

There was no monophyletic pattern of host or vector associations discernible in the phylogeny: species that are known to be associated with birds as reservoir hosts (i.e., *B. garinii*, *B. valaisiana*) did not cluster closely together but in different parts of the phylogeny suggesting that the host association has been acquired independently and not via the same common ancestor. This may explain why two species that apparently occupy the same ecological niche occur sympatrically. Both, *B. garinii* and *B. valaisiana* have been shown to utilize terrestrial birds as reservoir hosts and for both genospecies black birds and song thrushes play a prominent role as reservoir hosts [45, 46]. One conspicuous difference lies in the vector associations of these two species. While *B. valaisiana* is obviously transmitted by *I. ricinus* and its prevalence in Europe is as high as for *B. garinii* in some regions [47], *B. garinii* is also vectored by *I. persulcatus* [16, 39, 48, 49], *I. pavlovsky* [49, 50], and *I. uriae*, the latter a tick species that is adapted to sea birds [51–53]. Thus, apart from circulating in terrestrial transmission cycles, *B. garinii* is also maintained in sea bird transmission cycles by *I. uriae* [54–57]. An evolutionary scenario that might explain sympatry of the two European bird associated *Borrelia* genospecies is an overlap of sea-bird and terrestrial transmission cycles in northern Europe with a potential entrance port of *B. garinii* into terrestrial bird transmission cycles [54]. A different possibility is a rodent to bird host switch from *B. bavariensis* [58]. Investigating the underlying molecular mechanisms will help to elucidate how the host associations in these tick-borne microorganisms did evolve.

The same appears to be true for vector associations. The genospecies *B. garinii* or *B. burgdorferi* s.s. are associated with a large number of vector species (*B. garinii* – *I. ricinus, I. persulcatus, I. pavlovskyi, I. uriae; B. burgdorferi* s.s. – *I. ricinus, I. hexagonus, I. scapularis, I. pacificus, I. affinis*; for sake of clarity we were unable to add all known vector species in Fig. 1). Other Borrelia species show a narrow vector association, e.g., *B. spielmanii*, which has so far been found only in *I. ricinus* and occasionally in *I. hexagonus* [59]. *B. valaisiana* has mainly been found in *I. ricinus* and infection prevalence in *I. persulcatus* is drastically reduced compared to *I. ricinus* [43, 60]. In Asia, there was one report of *B. valaisiana* in *I. columnae* in Japan [16] and for one strain from Russia the vector is unclear, it could be *I. persulcatus* or *I. pavlovskyi* (see pubmlst.org/borrelia/). *Borrelia* strains that were initially thought to be *B. valaisiana* [61] were later shown to belong to a rodent adapted genospecies, *B. yantzensis* [3, 62, 63]. It makes an interesting parallel to *B. garinii* / *B. bavariensis* and provides additional evidence that host adaptation is a driver for the evolution of *Borrelia* genospecies. The data presented here also support the notion that vector adaptations determine the geographic distribution range of species. For example, genospecies such as *B. garinii* or *B. burgdorferi* s.s. have a huge geographic distribution range due to their many vector associations while others have much narrower distribution ranges (e.g., *B. japonica*, *B. tanukii*, *B. spielmanii*, or *B. californiensis*) [10, 11, 64–67].

It is much more difficult to make inferences about human pathogenicity because it is unknown whether species that are maintained in specific enzootic transmission cycles are actually human pathogenic or not, i.e., they are not being found in human patients because they are associated with vectors that do not bite humans at any frequency.

### Recombination, mutation and selection as drivers of the *B. burgdorferi* s.l. species complex

Recombination analysis using ClonalFrameML [32] showed a *R/θ* (recombination/mutation rate) value of 0.182 for our dataset. This suggests that, for the genes analyzed in this study, mutation was the leading force of diversification which was also the case for the main chromosome of *B. bavariensis* [31]. It supports our hypothesis that the use of chromosomal orthologous genes is a good strategy to reconstruct the main phylogeny of *Borrelia*, avoiding recombining loci that may bias phylogenetic inferences [68, 69]. Other authors have calculated elevated recombination compared to mutation; for example Jacquot et al. [20] quantified the recombination to mutation rates to 1.7 and Qiu et al. [70] found recombination to mutation rates of 3:1. However, in both studies plasmid sequences were included in the analysis which may have led to calculation of higher recombination rates than mutation rates. Interestingly, the same ClonalFrameML [32] analysis performed on the phylogeny including the outgroup resulted in a *R/θ* of 0.014. This ten times lower estimate of recombination to mutation rates when including *B. duttonii* can be explained by a very low (or even absence of) recombination between *Borrelia* of the LB group and the relapsing fever group.

The larger sample sizes available for four species (*B. burgdorferi* s. s., *B. garinii*, *B. bavariensis* and *B. afzelii*) allowed us to estimate within-species summary statistics (Table 1). We found negative values for Tajima’s *D* [34] for the whole dataset as well as in each of the four species. This is consistent with previous data for chromosomal regions of *B. burgdorferi* s. s., *B. garinii*, *B. afzelii* [20] and *B. bavariensis* [31] and suggests population expansions. Hypotheses of population expansion have been put forward by different studies using other methods with European *Borrelia* genospecies [7] and *B. burgdorferi* s.s. from the USA [71]. Another possible interpretation of negative Tajima’s *D* is strong purifying selection. However, Tajima’s *D* was variable between genes and we identified in each genospecies several genes that show extremely high or low values (more than two standard deviation from the mean, see Additional file 1: S4). These genes could have been the target of natural selection within the evolution of these genospecies. It is interesting to note that only five genes showed extreme values in two different genospecies suggesting that the four species have been under divergent selective processes. Genetic diversity (*π*) [33] was comparable in all four species and of the same order as in previously published chromosomal data [20, 31]. Only one gene showed extremely low diversity (BB0274 (*fliQ*) in *B. garinii*, Additional file 1: S4) and might have been under strong negative selection. Genes showing extreme high values of *π* (Additional file 1: S4) could be pseudogenes or under balancing selection (in particular if they also show high Tajima΄s *D*).

### Evaluating the explosive radiation hypothesis

It had been suggested that, in the evolutionary history of the *B. burgdorferi* s.l. complex, an “explosive radiation” was followed by a sharp decrease in diversification rates [18]. These authors reconstructed phylogenies for three genes using strains from 15 *Borrelia* species or seven genes using 11 *Borrelia* genospecies and concluded that this group had experienced an explosive radiation (star-like shape of the tree). Our phylogeny reconstruction does not support this hypothesis and the evolution rates in our phylogeny were not higher for the inner nodes as found by Morlon et al. [18]. Rough estimations of relative divergence of clades suggest that recurrent speciation has occurred within the *Borrelia burgdorferi* species complex. This discrepancy might be due to the low number of genes used by Morlon et al. [18]. Indeed if few genes are used and if their individual phylogenies are contradictory (due to recombination), a star-like shaped tree can result from the combination of the genes [72]. To test this we repeated the study by downloading from GenBank the sequences for the same three genes for 12 Borrelia species (see Additional file 1: S5 for detailed Methods). The phylogeny reconstructed using BEAST [26] for the three genes together was indeed star-like, but each individual phylogeny did not have the same shape and the three topologies differed (see Additional file 1: S7). To further test whether recombination between the genes was important, we used the four -gamete condition [73] to estimate recombination within and between genes (see Additional file 1: S6). No permutation of the SNPs positions between genes showed a lower ratio of within versus between genes recombination (see Additional file 1: for Methods), hence, showing that recombination within genes was significantly lower than between them. These results suggest that the star-like shape of the phylogeny reconstructed by Morlon et al. (2012) [18] was due to recombination between loci. Thus, we cannot confirm that the evolutionary history of *B. burgdorferi* sensu lato results from explosive radiation. In fact, we see that diversification and speciation events have been going on throughout the history of the complex.

### Where is the origin of Borrelia?

As already mentioned above, the phylogeny divides the genospecies into two major sister clades: one that harbors species known to occur in Europe and Asia and one that harbors species known from North America and Europe. At present, it is difficult to pinpoint the “origin” of the whole species complex.

Considering the “American Clade”, it seems that host and vector adaptations followed by diversification have led to speciation in North America [10, 74, 75]. There are two groups in the “American” cluster, *B. burgdorferi* s.s. and *B. bissettiae,* that occur in Europe as well as in North America. With regard to the origin of the species *B. burgdorferi* s.s., a North American and a European origin have been suggested [76, 77]. Interestingly, in the branch that leads to *B. burgdorferi* s.s., strains comprising the species *B. americana* have to date only been reported in North America [10, 74] while strains belonging to the divergent *B. burgdorferi* s.s. group (e.g., Z41293, branching off a little before the group at the tip) have been found only in Europe [76]. It poses the question whether the common ancestor of this clade originated in North America or Europe. It is reasonable to assume that either these species exist at such low frequency that they have not been detected on the respective continents so far or that some lineages may have died out on one or the other continent leading to the mosaic pattern of geographic occurrence of species observed today and therein lies the difficulty to obtain evidence for one or the other way.

## Conclusions

In conclusion, we suggest that the *B. burgdorferi* sensu lato complex represents a dynamic system resembling the cohesion ecotype model [78]. The data presented in our study show that switches of host associations followed by speciation have developed several times independently during the evolutionary history of the species complex and this dynamic interplay is likely still going on today. Recognition of diversification and speciation through vector adaptation, although known from the relapsing fever group of spirochetes, is new to the Lyme Borreliosis group of spirochetes with immense impact on the geographic distribution of species. We suggest that, apart from speciation via host association, diversification and speciation through vector adaptation may represent a general mechanism for diversification of tick-borne pathogens with potentially huge impact in the geographic distribution, as it may allow for invasion of new territories. The molecular mechanisms involved in host and vector switches deserve further investigation and the lists of genes showing extreme summary statistic values presented here are good candidates for such analyses.

## Methods

### Samples

We sequenced 93 Borrelia strains from 14 species of the *B. burgdorferi* s.l. complex (Additional file 1: S1). For most species only a limited number of strains was available but for four species we were able to include a larger number of strains to cover intraspecific variation. Strains were isolated between 1981 and 2011. Strain information, date of isolation, and geographic origin are given in Additional file 1: S1. Five reference sequences available in GenBank were included into the analysis: *B. burgdorferi* s.s. B31 (GenBank accession no. AE000783.1), *B. bavariensis* strain PBi (GenBank accession no. CP000013.1), *B. bissettiae* strain DN127 (GenBank accession no. CP002746.1), *B. valaisiana* strain VS116 (GenBank accession no. NZ_ABCY00000000.2), and *B. afzelii* strain PKo (GenBank accession no. CP000395.1) as well as 13 additional strains for which assembled chromosome data was available including *B. chilensis* strain VA1 (See Additional file 1: S1).

To generate genomic DNA for Illumina sequencing, *Borrelia* strains were cultured in MKP medium using conditions as described previously [79, 80]. Genomic DNA was extracted via a Maxwell® 16 using a Maxwell LED DNA kit (Promega, Germany). Following DNA quantification, libraries were prepared according to the Nextera DNA sample preparation guide (Illumina, San Diego CA, USA). The samples were diluted to a DNA concentration of 50 ng/μl and “tagmented” by simultaneously fragmenting DNA using transposomes as provided by the manufacturer and adding adapters. After tagmentation, samples having adapters on both ends underwent 5 PCR cycles to amplify the product and to add index primers. The resulting libraries were then validated using Agilent 2100 Bioanalyzer (Agilent, Germany).

### Sequencing and data processing

Sequencing was performed on an Illumina MiSeq platform (Illumina, San Diego CA, USA) that produced paired-end reads of 250 bp (Gene Centre, Laboratory for Functional Genome Analsis, LMU Munich; Source BioScience, Cambridge, UK). Some low quality samples were repeated on an Illumina HiSeq platform producing 100 bp long paired-end reads (Source BioScience). We thus had either HiSeq or MiSeq paired-end reads for each sample.

De novo assembly and alignment of contigs to the main chromosome of the closest related reference sequence from GenBank (see Additional file 1: S1) was performed in Galaxy [81–83] using the procedure as described elsewhere [31]. For each strain assembly was performed using two different assemblers; SOAPdenovo v1.0.0 [84] and VelvetOptimiser v1.0.0 [85] and data from both were merged into a single pileup file, if the SNPs identified with respect to the reference did not differ by more than 85 %. In case of higher differences, the data assemblies were uploaded into Integrative Genome Viewer (IGV) [86] to assess the differences between assemblies. In cases where one assembler had failed to generate matching contigs for one or several regions (creating a lot of incorrect SNPs) only the data from the assembler producing the better contigs was kept for analysis (Additional file 1: S1). Finally a FASTA file containing the data aligned to the relevant reference was created for each strain using home-made scripts that are available upon request. Data have been submitted to the SRA in GenBank.

### Selection of orthologous genes

The sequences of all open reading frames present in the reference sequence of *B. burgdorferi* s.s. strain B31 were downloaded from GenBank. This list of 1640 genes (including 840 genes annotated on the main chromosome) was then compared (blastn v.2.2.26, [25]) to the 93 sequence assemblies of strains sequenced by us and the 18 additional strains from GenBank. Only genes that were found once and only once with a score of at least 80 % similarity were kept for a reverse blast step were the sequence from the strain was compared to the B31 genes. Again only genes that had only one match with a similarity of at least 80 % for all strains were kept. In total, out of the 1,640 genes of reference B31we were able to identify 114 orthologous genes that were present in all strains.

Sequences from the 111 strains for these 114 genes were aligned using MUSCLE v3.8.31 [87].

The same procedure was repeated including the chromosome sequence of the relapsing fever *Borrelia duttonii* strain Ly which allowed to identify 37 orthologous genes for the LB strains and Ly. The data was again aligned using MUSCLE v3.8.31 [87].

### Phylogeny reconstruction

A combined phylogeny of the 114 genes for the 111 strains was reconstructed using BEAST v1.7.5 [26] using the following priors: GTR substitution model [88], estimated base frequencies, no Site Heterogeneity model, lognormal relaxed clock, coalescent model: speciation birth-death model under incomplete sampling [89] and UPGMA starting tree [90]. The Markov chain was run for 50,000,000 steps for three independent runs. The output was first examined using Tracer v1.6 [91] and a burn-in of 10 % was found appropriate for the first run and 30 % for the other two runs. A consensus tree was then reconstructed for each run using the 45,000 or 35,000 trees after burn-in using the software TreeAnnotator provided with BEAST [26]. The three trees were very similar and thus a good indicator that the program had converged. One of the trees is presented in Fig. 1. A phylogeny with the outgroup *B. duttonii* on 37 genes was reconstructed using the same procedure and priors. The chain was ran for 100 million steps and a consensus tree reconstructed without the first 10 % trees. All three repetitions reconstructed very similar phylogenies and one of them is presented in Additional file 1: S2.

We also used ClonalFrameML v2.01 [32] to reconstruct a phylogeny taking into account recombination. As recommended by the authors, the program was first run using a standard model were recombination parameters were shared between branches. The transition/transversion ratio was set to 5.47 (mean ratio estimated on the 114 genes separately) and we performed 100 pseudo-bootstrap replicates. The resulting estimates for the ratio of recombination over mutation (*R/θ* = 0.00412), inverse mean DNA import length (*1/δ* = 8.238 * 10–5) and mean divergence of imported DNA (*ν* = 0.0240) were then used in a subsequent run allowing for recombination rates to vary between branches with an allowed variability of recombination of 0.1. The same procedure was repeated for analysis based on the 37 genes with outgroup with a transition/transversion ratio set to 4.29. The standard model estimates *R/θ* = 0.00416, *1/δ* = 7.705 * 10–5 and *ν* = 0.0228 were again used in a subsequent run allowing for recombination rates to vary.

### Estimating divergence of clades

Without knowledge about mutation rates or generations per year, it is extremely difficult to accurately estimate the divergence of clades. To obtain a rough estimate of the relative time of splits between species, we used the following strategy: the phylogeny branch length are expressed in substitutions per site, so multiplying by a plausible mutation rate per generation allows a rough estimation of evolutionary distance expressed in generations. Dividing this estimation by 2 gives an approximate time of divergence. Previous authors have used a mutation rate per generation of 10^7^ [20]. In order to make our data comparable, we employed the same mutation rate.

### Summary statistics

Summary statistics were computed on 114 genes using R packages adegenet [92, 93] and pegas [94] on all samples and separately within the species *B. burgdorferi* s. s., *B. garinii*, *B. bavariensis* and *B. afzelii* excluding one misclassified sample (PKl)*.* These included nucleotide diversity (*π*) [33] which was calculated taking into account only sites with no missing data and Tajima’s *D* computed without sites with alignment gaps [34] Mean and standard deviation values are presented in Table 1 and genes with estimated values diverging by more than two standard deviations from the mean are listed in Additional file 1: S3.

## Additional file

Additional file 1:
**S1.** Strains included in this study, date of isolation, biological source, MLST sequence type (ST), geographic origin and previously determined species designation. **S2.** Phylogenetic inference including outgroup *B. duttonii.* **S3.** Estimates of divergence in generations for *Borrelia* genospecies. **S4.** Genes showing nucleotide diversity (*π*) (Nei 1987) or Tajima’s *D* (Tajima 1989) values exceeding two standard deviations in each species with respect to the 114 genes under study. **S5.** Additional material and methods: Phylogeny reconstruction and recombination estimation for three genes in 12 reference *Borrelia* species. **S6.** Four-Gamete analysis of loci used by Morlon et al. [18]. **S7.** Phylogenies reconstructed with BEAST v1.7.5 of a) three genes studied by Morlon et al. [18] combined, b) *fla*, c) *groEL* and d) *rrs*. (PDF 460 kb)
