# Supplementary material for: Recurrent evolution of host and vector association in bacteria of the Borrelia burgdorferi sensu lato species complex
Source: BMC Genomics. 2016 Sep 15;17:734. doi: 10.1186/s12864-016-3016-4 (PMC5025617; doi:10.1186/s12864-016-3016-4)

Additional files – Becker et al.

Additional file S1. **Table S1: Strains included in this study, date of isolation, biological source, MLST sequence type (ST), geographic origin and previously determined species designation**. Data type refers to the sequencer that was used for generation of sequence reads. Abbreviation of assembler used: S = SOAPdenovo v1.0.0 (Li et al. 2009), V = VelvetOptimiser v 1.0.0 (Zerbino and Birney, 2008), B: both combined

Strain	Date of isolation	Source	MLST ST	Origin	Species	Data type	Assembler	Aligned to ref
PAlt	1988	human	71	Germany	B. afzelii	MiSeq	B	Pko
PBabu	2001	human	71	Germany	*B. afzelii*	MiSeq	B	Pko
PBec	1988	human	540	Germany	*B. afzelii*	HiSeq	B	Pko
PFes	1988	human	462	Germany	*B. afzelii*	MiSeq	B	Pko
PHam	1987	human	71	Germany	*B. afzelii*	HiSeq	B	Pko
PJe	1992	human	458	Germany	*B. afzelii*	MiSeq	S	Pko
PKl – likely misnamed	1993	human	71	Germany	*B. afzelii*	HiSeq	B	Pko
PKr	1992	human	347	Germany	*B. afzelii*	HiSeq	B	Pko
PMel	1990	human	71	Germany	*B. afzelii*	HiSeq	B	Pko
PObf	1998	human	71	Germany	*B. afzelii*	HiSeq	B	Pko
UO1				Germany	*B. afzelii*	MiSeq	B	PBi
PSto	1986	human	548	Germany	*B. afzelii*	HiSeq	B	Pko
UO5			547	Germany	*B. afzelii*	HiSeq	B	Pko
SCW41	1994/ 1995	*I. minor*	449	USA, SC	*B. americana*	MiSeq	B	B31
A104S	1996	human	84	Netherlands	*B. bavariensis*	HiSeq	B	PBi
A91S	1996	human	84	Netherlands	*B. bavariensis*	MiSeq	B	PBi
PBaeII	1990	human	84	Germany	*B. bavariensis*	HiSeq	B	PBi
PBar	1988	human	84	Germany	*B. bavariensis*	HiSeq	B	PBi
PBN	1999	human	84	Germany	*B. bavariensis*	HiSeq	V	PBi
DK6	1990	human	84	Denmark	*B. bavariensis*	HiSeq	B	PBi
PEi	1994	human	84	Germany	*B. bavariensis*	HiSeq	B	PBi
FujiP2		*I. persulcatus*	371	Japan	*B. bavariensis*	MiSeq	B	PBi
PHerI	1989	human	84	Germany	*B. bavariensis*	MiSeq	B	PBi
Hiratsuka		human	128	Japan	*B. bavariensis*	MiSeq	B	PBi
J14		human	371	Japan	*B. bavariensis*	MiSeq	V	PBi
J15		human	381	Japan	*B. bavariensis*	MiSeq	B	PBi
J20T		*I. persulcatus*	383	Japan	*B. bavariensis*	MiSeq	B	PBi
Konnai17_clone1	2011	*I. persulcatus*	131	Japan	*B. bavariensis*	MiSeq	B	PBi
PLad	2000	human	84	Germany	*B. bavariensis*	HiSeq	V	PBi
Lubl25	1995	human		Slovenia	*B. bavariensis*	MiSeq	B	PBi
N346		*I. persulcatus*	386	Japan	*B. bavariensis*	MiSeq	V	PBi

PNeb	1988	human	84	Germany	*B. bavariensis*	MiSeq	B	PBi
PNi	2000	human	84	Germany	*B. bavariensis*	MiSeq	B	PBi
NT24		*I. persulcatus*	371	Japan	*B. bavariensis*	MiSeq	B	PBi
PRab	1994	human	84	Austria	*B. bavariensis*	MiSeq	B	PBi
PRof	1989	human	84	Germany	*B. bavariensis*	MiSeq	B	PBi
PTrob	1988	human	85	Slovenia	*B. bavariensis*	MiSeq	B	PBi
PWin	1987	human	85	Germany	*B. bavariensis*	HiSeq	V	PBi
PZwi	1994	human	85	Germany	*B. bavariensis*	HiSeq	B	PBi
PGeb	1996	human		Germany	*B. bissettiae*	MiSeq	B	DN127
PAbe	1997	human	1	Germany/USA?	*B. burgdorferi ss*	*MiSeq*	B	B31
PAli	1994	human	1	Germany/USA?	*B. burgdorferi ss*	*MiSeq*	B	B31
PBre	1988	human	20	Germany	*B. burgdorferi ss*	*MiSeq*	V	B31
PDri	1988	human	24	Germany	*B. burgdorferi ss*	*MiSeq*	B	B31
PFheI	2010	human	21	Germany	*B. burgdorferi ss*	*MiSeq*	V	B31
PFheII	2010	human	21	Germany	*B. burgdorferi ss*	*MiSeq*	B	B31
PFi I	1985	human	284	Germany	*B. burgdorferi ss*	*MiSeq*	B	B31
PGl	1993	human	21	Germany	*B. burgdorferi ss*	*MiSeq*	B	B31
PHas	1992	human	1	Germany/USA?	*B. burgdorferi ss*	*MiSeq*	B	B31
PKlF II	1988	human	24	Germany	*B. burgdorferi ss*	*MiSeq*	B	B31
PKu	1996	human	20	Germany	*B. burgdorferi ss*	*MiSeq*	B	B31
PLue	1999	human	3	Germany/USA?	*B. burgdorferi ss*	*MiSeq*	B	B31
PMeh	1996	human	20	Germany	*B. burgdorferi ss*	*MiSeq*	B	B31
PMi	1994	human	20	Germany	*B. burgdorferi ss*	*MiSeq*	B	B31
PSst	1993	human	20	Germany	*B. burgdorferi ss*	*MiSeq*	B	B31
Z4/12/93	1995	*I. ricinus*	27	Germany	*B. burgdorferi ss*	*MiSeq*	B	B31
CA446	1995	*Dipodomys californicus*	447	USA, CA	*B. californiensis*	MiSeq	B	DN127
SCW-22	1994	*I. minor*	450	USA, SC	*B. carolinensis*	MiSeq	B	DN127
20047		*I. ricinus*	82	France	*B. garinii*	MiSeq	V	PBi
PBes	1989	human	251	Germany	*B. garinii*	MiSeq	B	PBi
PBr	1985	human ospA3	244	Germany	*B. garinii*	HiSeq	B	PBi
PFr	1995	human ospA3	244	Germany	*B. garinii*	HiSeq	B	PBi
PHc	1996	human ospA6	86	Germany	*B. garinii*	MiSeq	B	PBi
PHez	1994	human ospA6	86	Germany	*B. garinii*	MiSeq	B	PBi
HT59		*I. persulcatus*	367	Japan	*B. garinii*	MiSeq	B	PBi
UO4			86	Germany	*B. garinii*	MiSeq	B	Pko
J21		human	127	Japan	*B. garinii*	MiSeq	B	PBi
PKi	1992	human ospA8	245	Germany	*B. garinii*	MiSeq	B	PBi
Konnai20_clone1	2011	*I. persulcatus*	384	Japan	*B. garinii*	MiSeq	B	PBi
UO3	1994	human ospA		Germany	*B. garinii*	HiSeq	B	PBi
PLa	1988	human	33	Germany	*B. garinii*	HiSeq	B	PBi

		ospA8						
PLi	1988	human ospA5	246	Germany	*B. garinii*	HiSeq	B	PBi
PMa	1989	human ospA6	87	Jugoslavia	*B. garinii*	MiSeq	B	PBi
Malouvrh		human ospA6	180	Slovenia	*B. garinii*	MiSeq	B	PBi
PMe	1988	human ospA5	246	Germany	*B. garinii*	MiSeq	B	PBi
PMek	1998	human ospA3	576	Germany	*B. garinii*	HiSeq	B	PBi
PMit	1997	human ospA6	177	Germany	*B. garinii*	MiSeq	B	PBi
PNov	1990	human ospA6	180	Germany	*B. garinii*	MiSeq	B	PBi
NT31		*I. persulcatus*	134	Japan	*B. garinii*	MiSeq	V	PBi
POhm	1991	human ospA6	86	Germany	*B. garinii*	HiSeq	B	PBi
UO2				Germany	*B. garinii*	HiSeq	B	PBi
PSoR	1989	human ospA6	86	Germany	*B. garinii*	MiSeq	B	PBi
PStg	1996	human ospA6	179	Germany	*B. garinii*	MiSeq	B	PBi
PUl	1999	human opA6	578	Germany	*B. garinii*	MiSeq	B	PBi
H014		*I. ovatus*	453	Japan	*B. japonica*	MiSeq	B	Pko
25015	1991	*I. scapularis*	280	USA, NY	*B. kurtenbachii*	MiSeq	S	DN127
PoTiB2		*I. ricinus*	456	Portugal	*B. lusitaniae*	MiSeq	B	VS116
PHap	1989	human		Germany	*B. spielmanii*	MiSeq	B	Pko
PMai	1991	human		Germany	*B. spielmanii*	MiSeq	B	Pko
PMEW	1987	human		Germany	*B. spielmanii*	HiSeq	B	Pko
100B40			213		*B. valaisiana*	MiSeq	B	VS116
89B13			97		*B. valaisiana*	MiSeq	B	VS116
Okinawa-CW62T	2004/2005	*Crocidura watasei*	322	Japan	*B. yangtzensis*	MiSeq	V	VS116
**Reference strains**							**GenBank accession number**	
B31	1981	*I. scapularis*	1	USA, NY	*B. burgdorferi ss*		AE000783.1	
DN127		*I. pacificus*	272	USA, CA	*B. bissettiae*		CP002746.1	
VS116		*I. ricinus*	95	Switzerland	*B. valaisiana*		ABCY02000001.1	
PKo	1984	human	71	Germany	*B. afzelii*		CP002933.1	
PBi		human ospA4	84	Germany	*B. bavariensis*		CP000013.1	
**Additional strains from GenBank**							**GenBank accession number**	
CA382		*I. pacificus*	1	USA, CA	*B. burgdorferi ss*		CP005925.1	
ZS7		*I. ricinus*	20	Germany	*B. burgdorferi ss*		CP001205.1	
156a		human	4	USA, NY	*B. burgdorferi ss*		ABCV02000001.1	

N40	*I. scapularis*	19	USA, NY	*B. burgdorferi ss*	CP002228.1
JD1	*I. scapularis*	11	USA, MA	*B. burgdorferi ss*	CP002312.1
BgVir	*I. persulcatus*		Russia	*B. (garinii) bavariensis*	CP003151.1
SZ	*Dermacentor*		China	*B. (garinii) bavariensis*	CP007564.1
NMJW1	*I. persulcatus*		China	*B. (garinii) bavariensis*	CP003866.1
HLJ01	human		China	*B. afzelii*	CP003882.1
Tom3107	*I. persulcatus*		Russia	*B. afzelii*	NZ_CP009212.1
K78	human		Austria	*B. afzelii*	CP009058.1
Tom4006	*I. persulcatus*		Russia	*B. valaisiana*	NZ_CP009117.1
VA1	*I. stilesi*		Chile	*B. chilensis*	CP009910.1
**Outgroup strain**					
Ly	human		Tanzania	*B. duttonii*	CP000976.1

Additional File S2. Phylogenetic inference including outgroup *B. duttonii.*

The phylogeny reconstructed with BEAST v1.7.5 [26] is based on 37 orthologous single copy genes. The scale bar indicates substitutions per site.

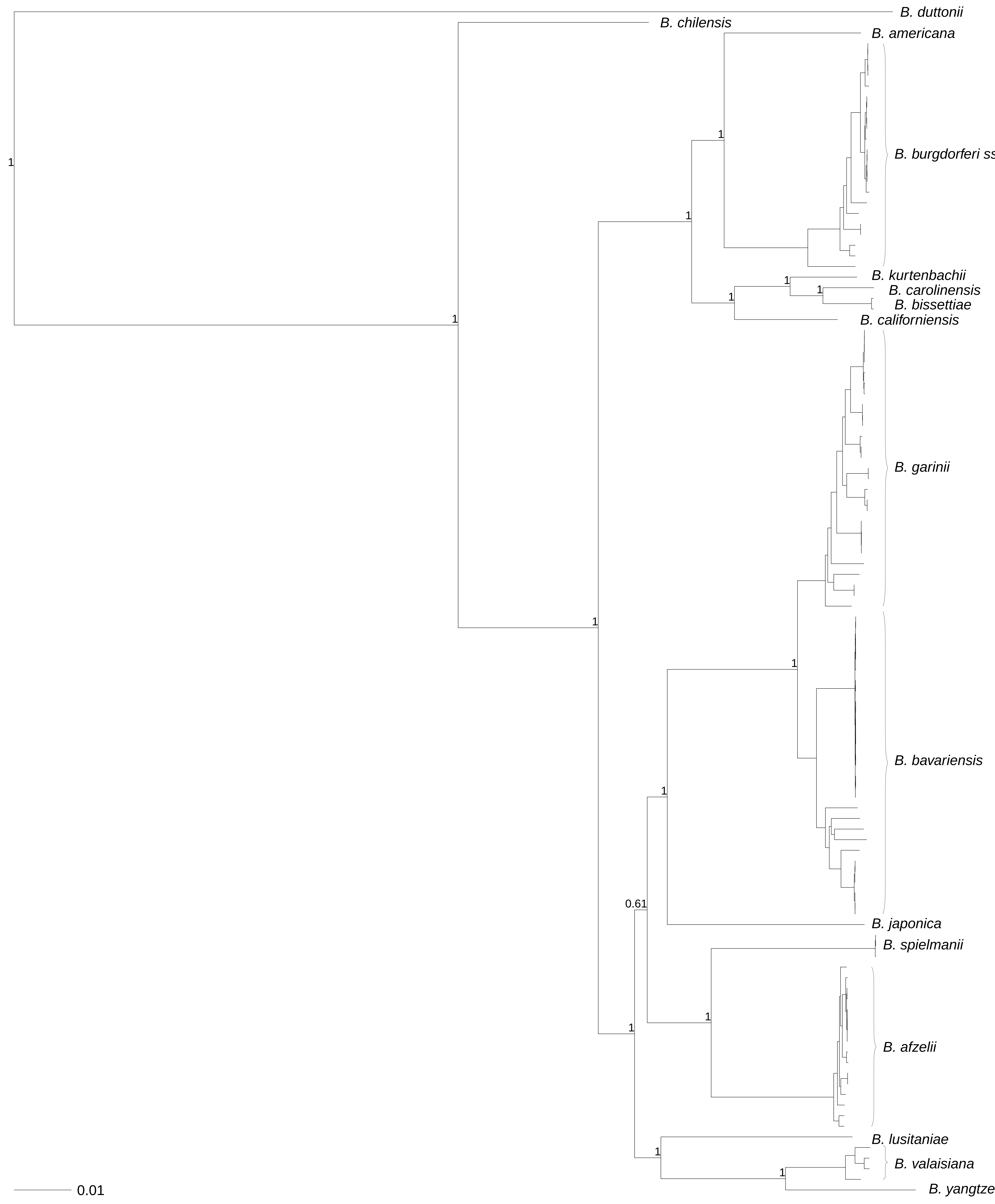
B. duttonii
B. chilensis
B. americana
B. burgdorferi ss
B. kurtenbachii
B. carolinensis
B. bissettiae
B. californiensis
B. garinii
B. bavariensis
B. japonica
B. spielmanii
B. afzelii
B. lusitaniae
B. valaisiana
B. yangtze
0.61
0.01

Additional file S3. Estimates of divergence in generations for *Borrelia* genospecies

Species	Divergence in
B. duttonii from LB	1,507,000
B. chilensis from other LB	639,000
B. garinii from *B. burdgorferi ss*	450,000
B. garinii from *B. bavariensis*	137,000
European from Asian *B. bavariensis*	92,000
B. burdgorferi ss from *B. americana*	253,000
B. burdgorferi ss from *B. californiensis*	291,000
B. burdgorferi ss from *B. carolinensis*	327,000
B. burdgorferi ss from *B. bissettiae*	323,000
B. burdgorferi ss from *B. kurtenbachii*	305,000

Additional file S4. Genes showing nucleotide diversity (*π*) (Nei 1987) or Tajima ´s *D* (Tajima 1989) values exceeding two standard deviations in each species with respect to the 114 genes under study. Low values are underlined and high values are in bold letters.

A) ***B. garinii***

gene	Diversity (*π*)	Tajima´s *D*	start	stop	name	function
BB0274	<u>0</u>		287910	288173	fliQ	flagellar biosynthesis protein
BB0047	0.005	**1.01**	46011	46379		
BB0483	0.005	**0.95**	499341	499703		50S ribosomal protein
BB0280	0.006	**1.13**	291644	292426	MotB	flagellar motor protein
BB0390	0.012	**0.80**	405243	405617	rplL	50S ribosomal protein
BB0485	**0.014**	**0.82**	500593	501009	rplP	50S ribosomal protein
BB0703	**0.019**	-0.20	740240	740422	rpmF	50S ribosomal protein
BB0618	**0.030**	**1.81**	645137	645601	cdd	cytidine deaminase

C) ***B. bavariensis***

gene	Diversity (*π*)	Tajima´s *D*	start	stop	name	function
BB0538	0.009	**1.20**	548487	548885		
BB0611	0.012	**1.57**	637972	638556	clpP	ATP-dependent Clp protease proteolytic subunit
BB0541	**0.024**	**1.61**	552007	552225		
BB0618	**0.034**	**1.52**	645137	645601	cdd	cytidine deaminase

C) ***B. burgdorferi*** **s.s.**

gene	Diversity (*π*)	Tajima´s *D*	start	stop	name	function
BB0480	0.002	**0.22**	497895	498191	rplW	50S ribosomal protein
BB0703	0.003	**0.22**	740240	740422	rpmF	50S ribosomal protein
BB0168	0.003	**1.03**	170720	171097		dnaK suppressor
BB0229	0.010	**0.12**	235289	235534	rpmE	50S ribosomal protein
BB0809	**0.010**	-1.95	854869	855996	tgt	queuine tRNA-ribosyltransferase
BB0570	**0.012**	-0.31	583458	583832		chemotaxis response regulator
BB0815	**0.013**	-0.46	861784	862677		
BB0123	**0.015**	**0.72**	119807	120589	rpsB	30S ribosomal protein

D) ***B. afzelii***

gene	Diversity (*π*)	Tajima´s *D*	start	stop	name	function
BB0293	0.001	**1.47**	302851	303309	flgC	flagellar basal body rod protein
BB0390	0.001	**1.47**	405243	405617	rplL	50S ribosomal protein
BB0487	0.002	**1.31**	501215	501469	rpsQ	30S ribosomal protein
BB0017	0.003	**1.70**	15995	16804		integral membrane protein
BB0570	**0.005**	**1.39**	583458	583832		chemotaxis response regulator
BB0538	**0.005**	-0.14	548487	548885		
BB0824	**0.005**	-0.70	868195	868740		
BB0131	**0.007**	0.33	126316	127413		recombinase A

Additional File S5. Additional material and methods

Phylogeny reconstruction and recombination estimation for three genes in 12 reference *Borrelia* species

Following Morlon et al. (2012) we dowloaded from GenBank sequences for 12 *Borrelia* species for the genes *fla*, *groEL* and *rrs*. Information on the strains chosen can be found in Table S2. Phylogenies for each of the three genes and combined phylogeny using the three genes were reconstructed using BEAST v1.7.5 (Drummond and Rambault 2007) with the same priors as for the main analysis on the 123 genes but using the HKY (Hasegawa et al. 1985) substitution model as this was chosen by Morlon et al. (2012). The reconstructed phylogenies can be seen on Figure S2.

The phylogenies reconstructed show a clear difference and the star-like shape of the combined tree might be due to the fact that the three genes show different phylogenies as recombination occurred between them. To test for this, we estimated recombination within and between genes using the four -gamete condition (Hudson and Kaplan 1985). We counted the number of pairs of SNPs within and between genes that had more than three allele combinations (indicates recombination under an infinite-sites model) in the 12 strains. The results can be seen in Table S3. We then permuted the SNPs within genes and computed the same analysis recording the following ratio using the number of pairs of SNPs having more or less than three allele combinations: $\dfrac{\dfrac{nb>3 withingenes}{nb>3 betweengenes}}{\dfrac{nb<4 withingenes}{nb<4 betweengenes}}$. None of the 1000 performed permutations had a lower ratio than the real dataset, showing that recombination within genes was significantly lower than between genes.

Reference not cited in the main paper:

Hasegawa M, Kishino H, Yano T. 1985. Dating of the human-ape splitting by a molecular clock of mitochondrial DNA. J Mol Evol. 22:160-174.

Additional file S6. Four-Gamete analysis of loci used by Morlon et al. 2012

	**WITHIN GENES**	**BETWEEN GENES**						
**gene(s)**	**4gamete violated**	**4gamete ok**	**% violated**	**4gamete violated**	**4gamete ok**	**% violated**	**length**	**nb snps considered**
**all within**	1028	8843	0,104	1552	7687	0,168		
**fla**	827	7951	0,094	1335	7044	0,159	1011	133
**groEL**	161	742	0,178	1160	5419	0,176	1638	43
**RRS**	40	150	0,211	609	2911	0,173	1539	20

Additional file S7. Phylogenies reconstructed with BEAST v1.7.5 (Drummond and Rambault 2007) of a) three genes studied by Morlon et al. (2012) combined, b) *fla*, c) *groEL* and d) *rrs*.

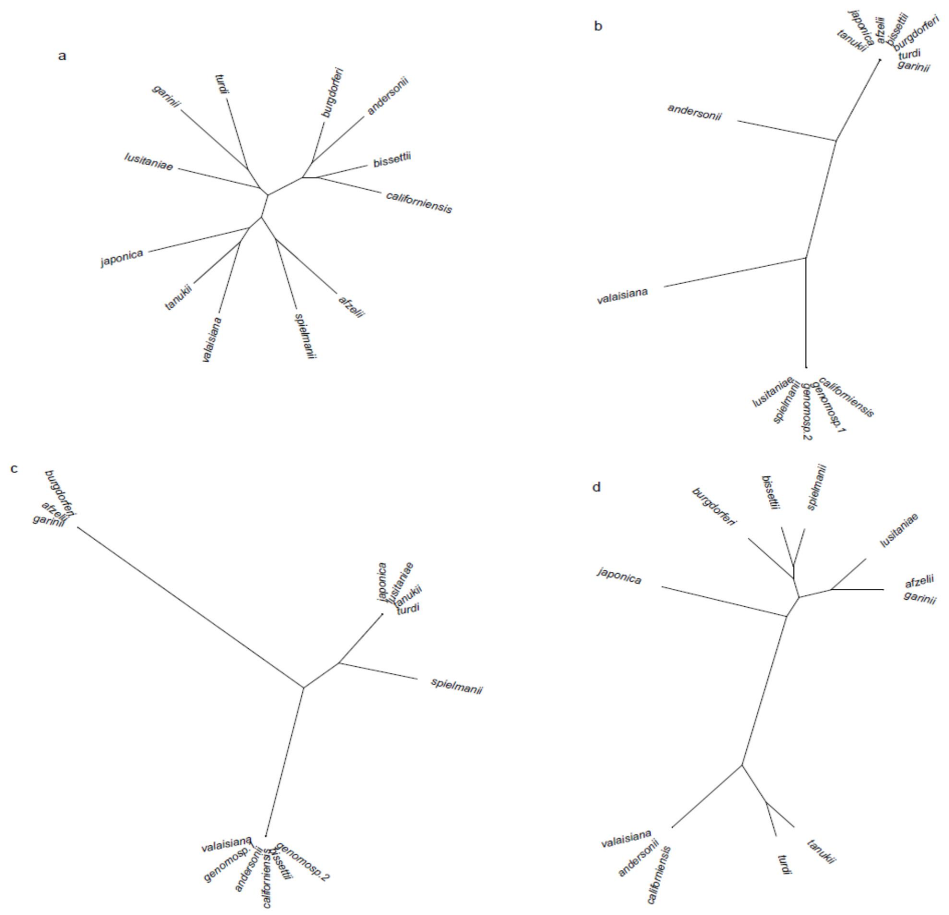

Supplement: Additional file 1: — S1. Strains included in this study, date of isolation, biological source, MLST sequence type (ST), geographic origin and previously determined species designation. S2. Phylogenetic inference including outgroup B. duttonii. S3. Estimates of divergence in generations for Borrelia genospecies. S4. Genes showing nucleotide diversity (π) (Nei 1987) or Tajima’s D (Tajima 1989) values exceeding two standard deviations in each species with respect to the 114 genes under study. S5. Additional material and methods: Phylogeny reconstruction and recombination estimation for three genes in 12 reference Borrelia species. S6. Four-Gamete analysis of loci used by Morlon et al. [18]. S7. Phylogenies reconstructed with BEAST v1.7.5 of a) three genes studied by Morlon et al. [18] combined, b) fla, c) groEL and d) rrs. (PDF 460 kb) [file 12864_2016_3016_MOESM1_ESM.pdf]
